# Development of Novel Single-Stranded Nucleic Acid Aptamers against the Pro-Angiogenic and Metastatic Enzyme Heparanase (HPSE1)

**DOI:** 10.1371/journal.pone.0037938

**Published:** 2012-06-15

**Authors:** Suzanne C. Simmons, Edward A. McKenzie, Lynda K. Harris, John D. Aplin, Paul E. Brenchley, Maria N. Velasco-Garcia, Sotiris Missailidis

**Affiliations:** 1 Department of Life, Health and Chemical Sciences, The Open University, Milton Keynes, Buckinghamshire, United Kingdom; 2 Manchester Interdisciplinary Biocentre, University of Manchester, Manchester, Metropolitan County of Greater Manchester, United Kingdom; 3 Maternal and Fetal Health Research Centre, University of Manchester, Manchester, Metropolitan County of Greater Manchester, United Kingdom; 4 Renal Research Group, University of Manchester, Manchester, Metropolitan County of Greater Manchester, United Kingdom; University of Helsinki, Finland

## Abstract

Heparanase is an enzyme involved in extracellular matrix remodelling and heparan sulphate proteoglycan catabolism. It is secreted by metastatic tumour cells, allowing them to penetrate the endothelial cell layer and basement membrane to invade target organs. The release of growth factors at the site of cleaved heparan sulphate chains further enhance the potential of the tumour by encouraging the process of angiogenesis. This leads to increased survival and further proliferation of the tumour. Aptamers are single or double stranded oligonucleotides that recognise specific small molecules, peptides, proteins, or even cells or tissues and have shown great potential over the years as diagnostic and therapeutic agents in anticancer treatment. For the first time, single stranded DNA aptamers were successfully generated against the active heterodimer form of heparanase using a modified SELEX protocol, and eluted based on increasing affinity for the target. Sandwich ELISA assays showed recognition of heparanase by the aptamers at a site distinct from that of a polyclonal HPSE1 antibody. The binding affinities of aptamer to immobilised enzyme were high (7×10^7^ to 8×10^7^ M^−1^) as measured by fluorescence spectroscopy. Immunohistochemistry and immunofluorescence studies demonstrated that the aptamers were able to recognise heparanase with staining comparable or in some cases superior to that of the HPSE1 antibody control. Finally, matrigel assay demonstrated that aptamers were able to inhibit heparanase. This study provides clear proof of principle concept that nucleic acid aptamers can be generated against heparanase. These reagents may serve as useful tools to explore the functional role of the enzyme and in the future development of diagnostic assays or therapeutic reagents.

## Introduction

Heparanase is a β-1,4-endoglycosidase enzyme [1] that participates in extracellular matrix (ECM) degradation and remodelling [1]. The nascent polypeptide is a 543 amino acid pre-proenzyme which, after removal of the signal peptide sequence in the endoplasmic reticulum, undergoes proteolytic processing in late endosomes/lysosomes by cathepsin-L like proteases [2] at sites Glu^109^-Ser^110^ and Gln^157^-Lys^158^, yielding an N-terminal 8kDa polypeptide, a C-terminal 50kDa polypeptide and between them; a 6kDa linker polypeptide [3]. The 50 and 8kDa polypeptides associate to form a heterodimeric active enzyme, whilst the 6 kDa linker is excised and degraded [3]. Heparanase activity is associated with activated leukocytes, mast cells, placental tissue and macrophages and the enzyme is secreted by activated CD4^+^ T cells [4], [5], [6], platelets [3], neutrophils and metastatic cells [7].

Upon secretion of heparanase from metastatic tumour cells, the enzyme hydrolyses the glycosidic bonds of heparan sulphate chains attached to proteoglycans to products 10–20 sugar units in length [8], leading to penetration of the endothelial cells of blood vessels and target organs by the tumour cell. Liberation of bound cytokines and growth factors sequestered by heparan sulphate chains in tissues [9] further facilitates growth of the tumour and promotes angiogenesis and proliferation of secondary tumours [10]. Levels of heparanase expression in tumour cells correlate with their metastatic potential; elevated levels of heparanase mRNA and protein have been found in cancer patients who show significantly shorter postoperative survival times than patients whose heparanase levels are normal [10], [11]. In addition to its function in cancer progression, heparanase enzyme also plays a major role in the activity of inflammatory cells. The enzyme has been detected in a variety of immune cells including T and B cells, macrophages, neutrophils and mast cells. It has been shown to mediate extravasation through the endothelial barrier via the remodeling of ECM heparan sulphate, which then allows trafficking to the sites of inflammation [7], [12], [13]. Heparanase expression has been linked to tumorigenesis in a number of different cancers, for example, acute myeloid leukaemia [14], bladder, brain [15], breast [16], colon [17], gastric [18], oesophageal [19], oral [20], and pancreatic [11], suggesting that it may be a suitable target for drug therapy. Currently available inhibitors of heparanase include neutralising antibodies [21], peptides [22] and small molecules [23], [24] as well as heparin [25] and sulphated oligosaccharide mimics of heparan sulphate [26], [27].

Aptamers are short DNA or RNA oligonucleotides developed for diagnostic and therapeutic use that display high binding affinity and specificity for target molecules [28], [29], [30]. The affinity of aptamers has been compared with that of antibodies (i.e. in the nanomolar range), but as aptamers are mostly smaller (8–25kDa compared to 150kDa), they can both penetrate tissues and be cleared from the plasma within minutes of intravenous administration, without triggering an immune response, which can be useful when using them as diagnostic agents [31]. For therapeutic use they are able to retain their function and binding characteristics upon modification with other moieties to improve their stability and solubility, whilst reducing their toxicity and plasma clearance [31], [32], [33], [34], [35], [36], [37]. Typically, aptamers are from 22 to 100 bases in length, and contain a region of variable sequence, flanked by known sequences, which are used for amplification and identification purposes. A large repertoire of different sequence combinations (typically in the region of 10^15^) in the central domain creates many different folding arrangements, specificities for different molecules and binding affinities. Aptamers are typically produced based on the SELEX (systematic evolution of ligands by exponential enrichment) procedure [38], although a number of other selection methodologies are currently available [39], [40], [41].

In this study, aptamers displaying high affinity and specificity were generated against the active enzyme heparanase using a modified SELEX procedure. Their binding to heparanase was characterised using ELISAs, quartz crystal microbalance (QCM) and fluorescence quenching experiments and aptamers were found to interact with the enzyme with equilibrium association constants in the range of 10^7^ M^−1^ and could detect heparanase at 10 nM in buffer. Aptamers bound to the enzyme at a site different to that recognised by the antibody used as a control, but in cell and tissue labelling studies they demonstrated comparable and in one instance superior recognition and staining, as well as the ability to inhibit heparanase action in matrigel assays. Thus, aptamers have shown the ability to specifically recognise purified heparanase as well as heparanase expressed in cells and tissue, offering the potential for further therapeutic and diagnostic development.

## Results

### Selection Generated Potentially High Affinity Aptamer Species

**Figure 1:**
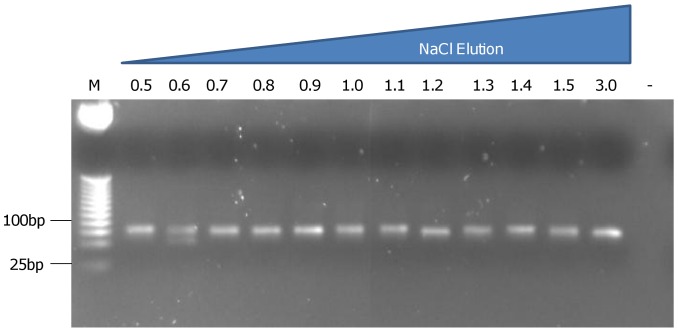
A 2% agarose gel to determine the presence of aptamers in each salt elution from 0.5 M NaCl to 3.0 M NaSCN. Bands at 75bp were observed in all fractions except the negative control, suggesting that different species of aptamer had bound with different affinities, removed in each salt concentration.

Aptamers were selected against heparanase using a modified single-round SELEX protocol. Briefly, the aptamer library was incubated with heparanase in a Top Yield PCR tube for one hour at room temperature and eluted using a salt step gradient. Eluates were desalted using Microcon filters and amplified by PCR. Aptamers were detected by agarose gel electrophoresis (figure 1) in all salt fractions. The 1.5 M NaCl and 3.0 M NaSCN elutions were selected for cloning as they were expected to contain aptamers with the highest affinity for the target. After sequencing, 81% of clones from the 3.0 M NaSCN elution had identical sequences, whilst a lower consensus was exhibited in clones from 1.5 M NaCl elution, with 2 identical species and areas of similarity in many others (table 1).

**Table 1:** Sequence consensus of aptamers eluted from 1.5 M NaCl and 3.0 M NaSCN.

1.5 M NaCl selection	3.0 M NaSCN selection
--------AAGAAGACTAGCGATGAAGAGCGCT-- 25CGGATTATTCGAACTCTTCCAATGA------------ 25----TACTAACTACTCGTACGCAAACGGA------- 25-----AATAGCAAGTTGTAAGTTGAAGAAA----- 25---ACTTAATTTGCACTTACCATATGGC--------- 25--TAATTGCTTCACCCTTACAAACCAG---------- 25-TCTCTTTCTTGACGCTCTATAAGCG----------- 25GCGATCGGGATAATCTGCTATAAGA---------- 25-------TATTCATACG—GAACAGAATTCTCCC- 25---------CCAATATAA-GAATATTATTGTCTCA- 25-----CATTGGAAAAATCCGAAAGTGATTA----- 25-----CAGATTAACATACCAAATCTAATTA----- 25---------ATGGACTTTTGAATGTGGCAACAAA- 25---------ATGGACTTTTGAATGTGGCAACAAA- 25---------GTCGCCTTTTCACTTCACTAGGGTA- 25GGACAGTCAGTCACCATGATTATTT---------- 25-------CACTCAACAGCCTTATTTCCCAATG--- 25-----GAAAGCAAACGTCCTAATTCCAGAA--- 25-------AACTAAATCTCCAAACAACTTACTG— 25-CACCAGGTTCCATGTCCTAAACTTA--------- 25-------TAATACCTCGCCAGCTACGTCACAG--- 25	TTTAACGTATTTATTCAAGCTCGTA--- 25TTTAACGTATTTATTCAAGCTCGTA--- 25TTTAACGTATTTATTCAAGCTCGTA--- 25TTTAACGTATTTATTCAAGCTCGTA--- 25TTTAACGTATTTATTCAAGCTCGTA--- 25TTTAACGTATTTATTCAAGCTCGTA--- 25TTTAACGTATTTATTCAAGCTCGTA--- 25TTTAACGTATTTATTCAAGCTCGTA--- 25TTTAACGTATTTATTCAAGCTCGTA--- 25TTTAACGTATTTATTCAAGCTCGTA--- 25TTTAACGTATTTATTCAAGCTCGTA--- 25TTTAACGTATTTATTCAAGCTCGTA--- 25TTTAACGTATTTATTCAAGCTCGTA--- 25TTTAACGTATTTATTCAAGCTCGTA--- 25TTTAACGTATTTATTCAAGCTCGTA--- 25TTTAACGTATTTATTCAAGCTCGTA--- 25TTTAACGTATTTATTCAAGCTCGTA--- 25TTTAACGTATTTATTCAAGCTCGTA--- 25TTTAACGTATTTATTCAAGCTCGTA--- 25TTTAACGTATTTATTCAAGCTCGTA--- 25TTTAACGTATTTATTCAAGCTCGTA--- 25TTTAACATATTTATTCAAGCTCGTA--- 25---TATATATTCCTACACATCTAACTTA 25

**Figure 2:**
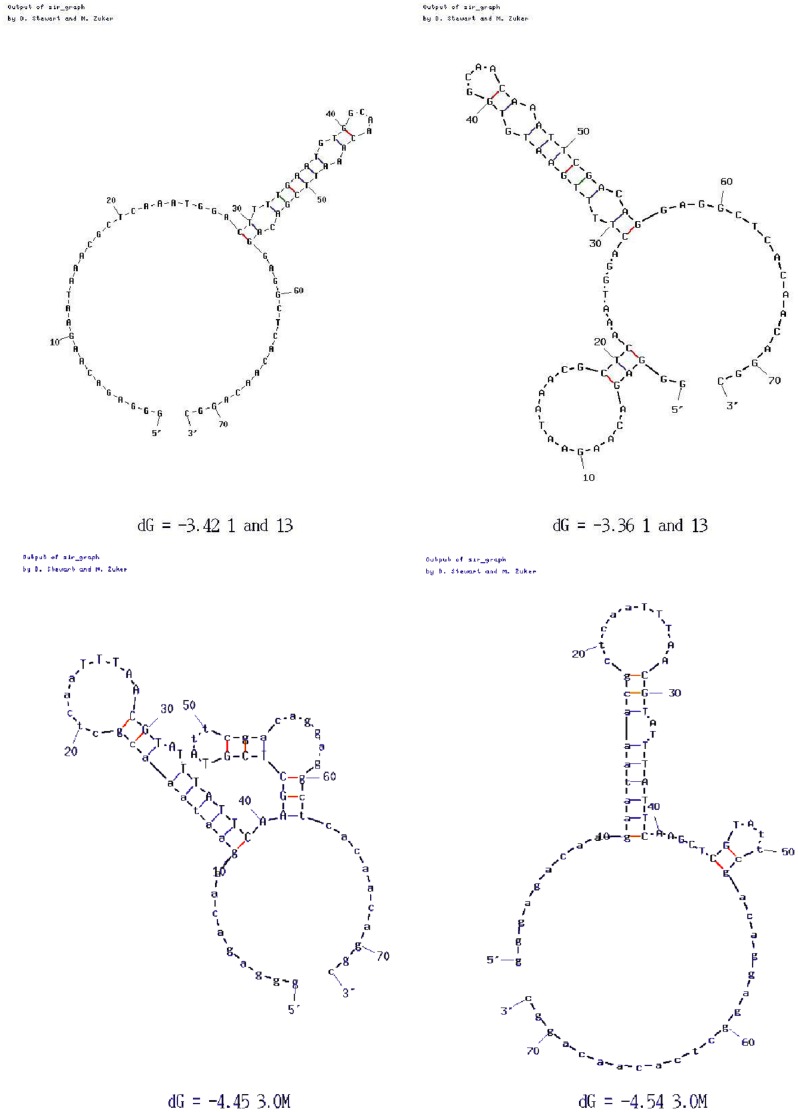
Mfold predicted structures for products of 1.5 M NaCl (above) and 3.0 M NaSCN (below) selection.

The homologous aptamer sequences from both 1.5 M NaCl and 3.0 M NaSCN elutions were analysed using the Mfold [42], [43] program (figure 2) which predicts three-dimensional structures by calculating their minimum Gibbs free energies as well as the free energy of folding of each base pair at 37°C. Both the variable and primer regions were analysed, as the primer regions often help to confer stability upon the aptamer and are therefore frequently implicated in their structure. Based on the structure adopted by the identical species in the 1.5 M NaCl elution, a truncated 30 nucleotide sequence, predicted to form a stem loop; ‘1.5 M short’, and the 73 nucleotide full length sequence; ‘1.5 M long’ were synthesised for further testing. The sequence generated from 3.0 M NaSCN was synthesised as 55 nucleotides ‘3.0 M’, due to this number of nucleotides being consistently implicated in the predicted structures.

### Aptamer Species Bind with High Affinity to the Target, Heparanase

**Figure 3:**
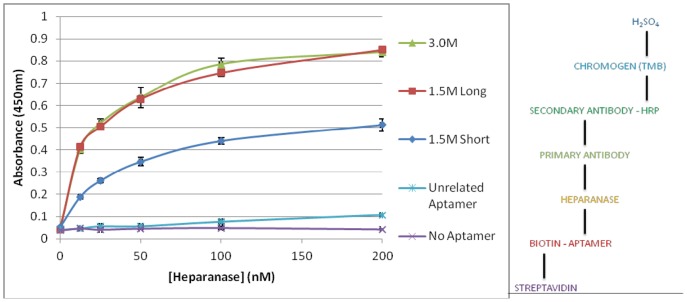
Streptavidin ‘sandwich’ ELISA testing binding of 187 nM ‘1.5 M short’, ‘1.5 M long’, ‘3.0 M’ and an unrelated aptamer to heparanase, with a no aptamer negative control. Schematic diagram (right) of the ELISA.

Competition ELISAs determined that a heparanase polyclonal antibody and the selected aptamers do not compete for the same binding site (data not shown) as there was no reduction in signal from the polyclonal antibody upon aptamer addition. Therefore, their binding was investigated by a sandwich ELISA, where both antibody and aptamer were utilised (figure 3). 5′-biotinylated aptamers were immobilised via streptavidin to the plate surface. After incubation with increasing concentrations of heparanase, then successive incubations with polyclonal antibody, horseradish peroxidase conjugated secondary antibody and addition of chromogen and stop solution, an increase in signal corresponding with the increase in heparanase concentration was observed. An unrelated aptamer generated a low background signal only, confirming specific binding of the selected aptamers to heparanase. The graph of 1.5 M long and 3.0 M aptamers shows a plateau at 100 nM heparanase, with a plateau at 70 nM for 1.5 M short aptamer, when binding sites become exhausted. Also, the absorbance reaches a higher maximum for the two longer aptamers which would suggest that more heparanase is retained by them. This may be explained by their size, in that more 9kDa 1.5 M short aptamers may be obscured and be subject to steric hindrance by one 58kDa heparanase molecule than the 22kDa 1.5 M long or 17kDa 3.0 M aptamer.

**Figure 4:**
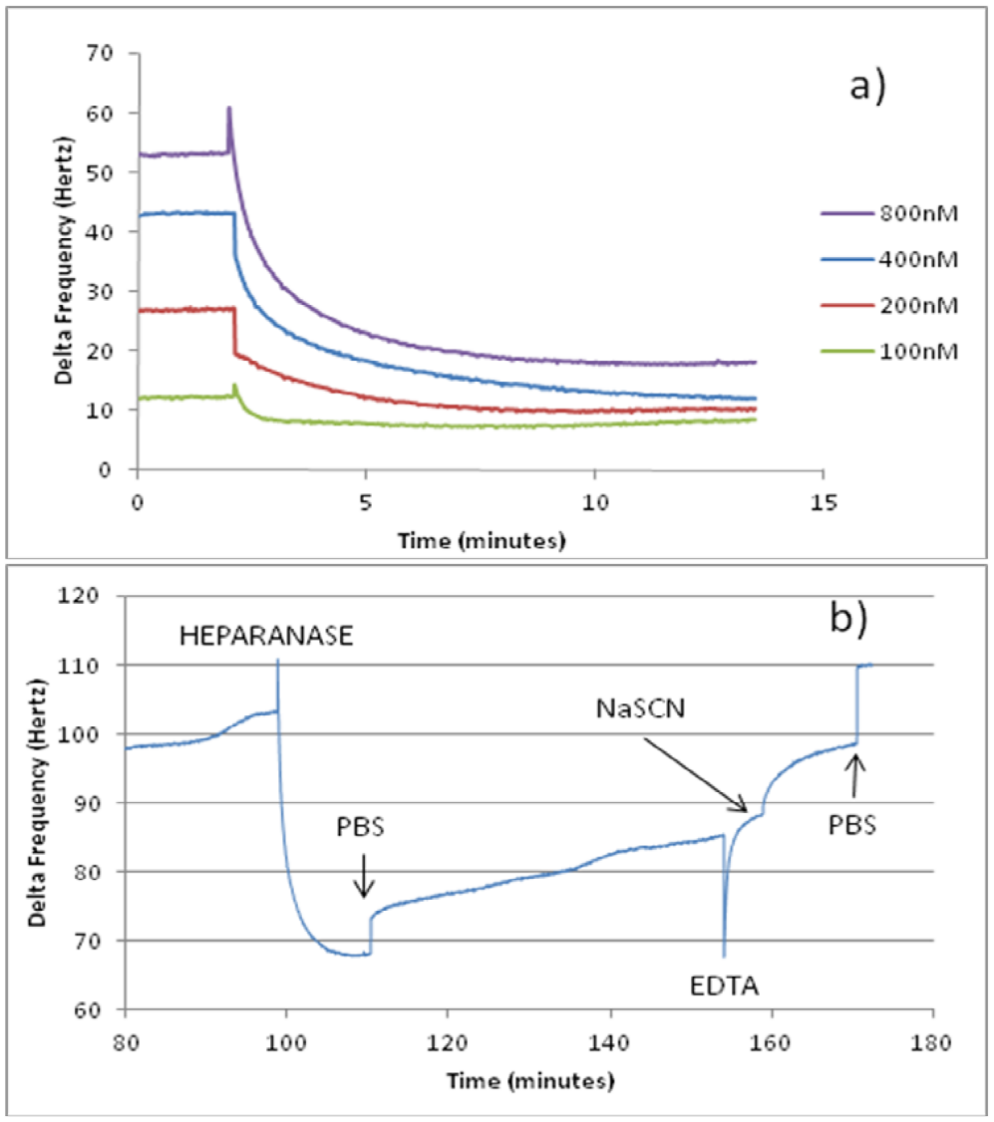
Binding sensogram (a) showing decrease in frequency of crystal immobilised with 1.5 M short aptamer exposed to different concentrations of heparanase. Higher concentrations of heparanase exhibited a greater decrease in frequency. Binding and regeneration sensogram (b) for addition of 800 nM heparanase to a quartz crystal immobilised with 1.5 M short aptamer. After a PBS wash, a Δfr of 37.46 Hz was observed, confirming heparanase was retained and bound. Washing with EDTA and 3.0 M NaSCN allowed almost total regeneration of the crystal.

Aptamers were subsequently used to capture and bind heparanase from solution (100–800 nM) in QCM experiments. Figure 4 shows the sensograms obtained for aptamer 1.5 M short and confirms a decrease in frequency upon addition of heparanase, which only reverses marginally after the PBS wash; generating a resonance frequency decrease of 37.46Hz, thus suggesting binding of heparanase. Upon addition of heparanase, the resonance frequency declined in a dose-dependent manner. Baseline characteristics were restored by exposure to EDTA and 3.0 M NaSCN, consistent with desorption.

**Figure 5:**
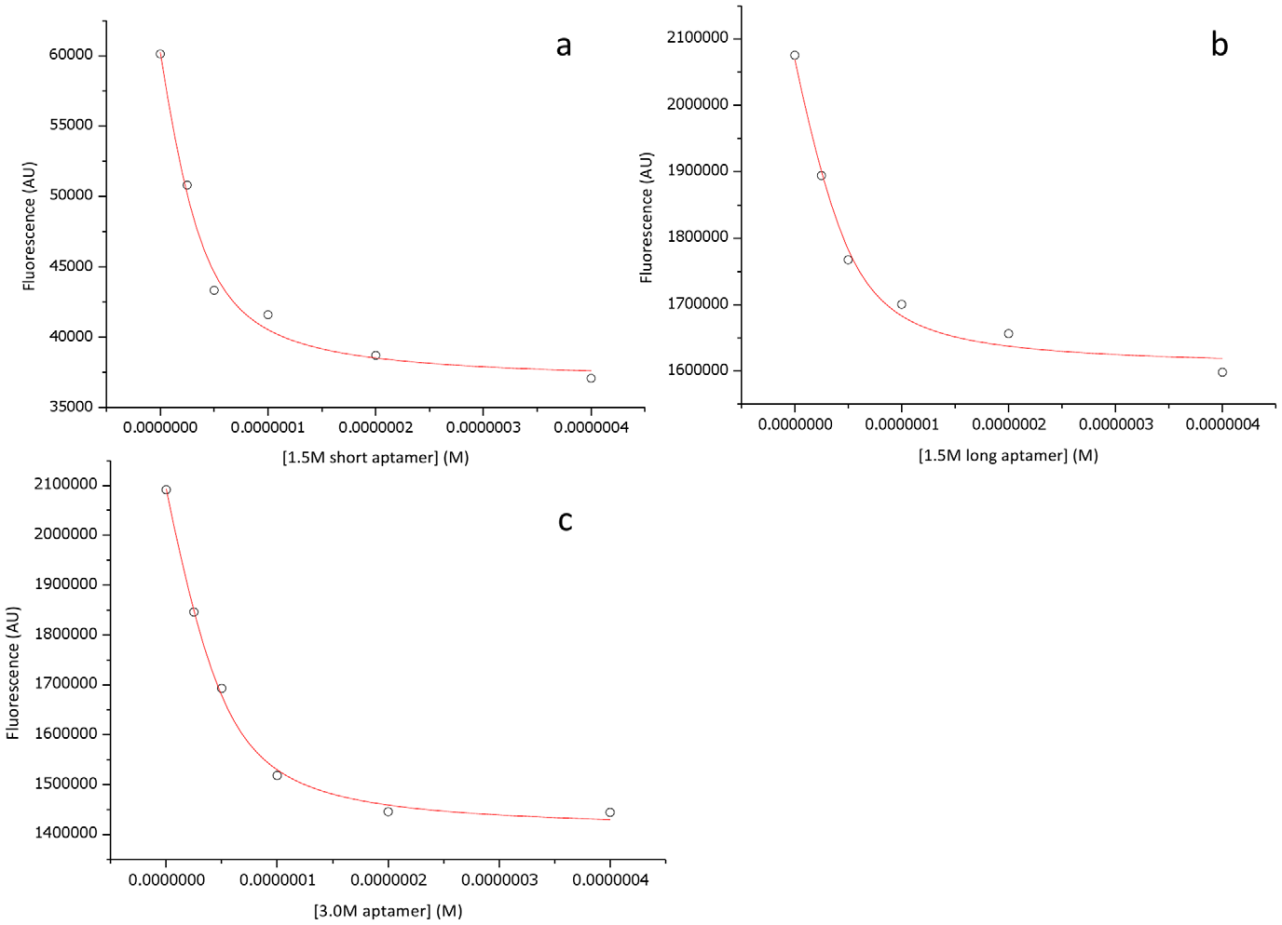
Fluorescence quenching titrations of 250 nM heparanase by 1.5 M short (a), 1.5 M long (b), and 3.0 M NaSCN (c) aptamers.

Fluorescence quenching titrations were carried out using the natural intrinsic fluorescence of heparanase (due to the solvatochromic emission peak of tryptophan) to determine the binding interactions of the three aptamers, analysed by a quadratic equation [44]. Figure 5 shows quenching of the maximal fluorescence of heparanase with 1.5 M short, long and 3.0 M aptamers by 38%, 23% and 31% respectively. Equilibrium association constants for 1.5 M short, 1.5 M long and 3.0 M aptamers were calculated to be 7.3, 8.3 and 7.6×10^7^ M^−1^ respectively, suggesting that although the primer regions included in the 1.5 M long sequence do appear to have an effect on the interaction with heparanase, this is not a significant one. The quenching percentage from the heparanase-selected aptamers suggest they are binding further from a region containing one or more of the six tryptophan residues. As all tryptophans are spread within the 50kDa subunit at sites 199, 220, 295, 340, 365 and 405, it is likely that the binding of the aptamer would be closer to one or two tryptophans at a time, clearly accounting for the percentage of change observed in the fluorescence emission. It is even possible that the aptamer binds to the 8kDa subunit (which contains no tryptophan residues) or N-terminus, which carries a positive charge at physiological pH so would attract the negatively charged aptamer, but the aptamer binding to this part of the heterodimer causes such changes in the structure that they would affect the overall fluorescence emission of the tryptophans in the 50kDa subunit.

### Aptamer Species Bind Heparanase with High Affinity and Specificity in Cell and Tissue Studies

**Figure 6:**
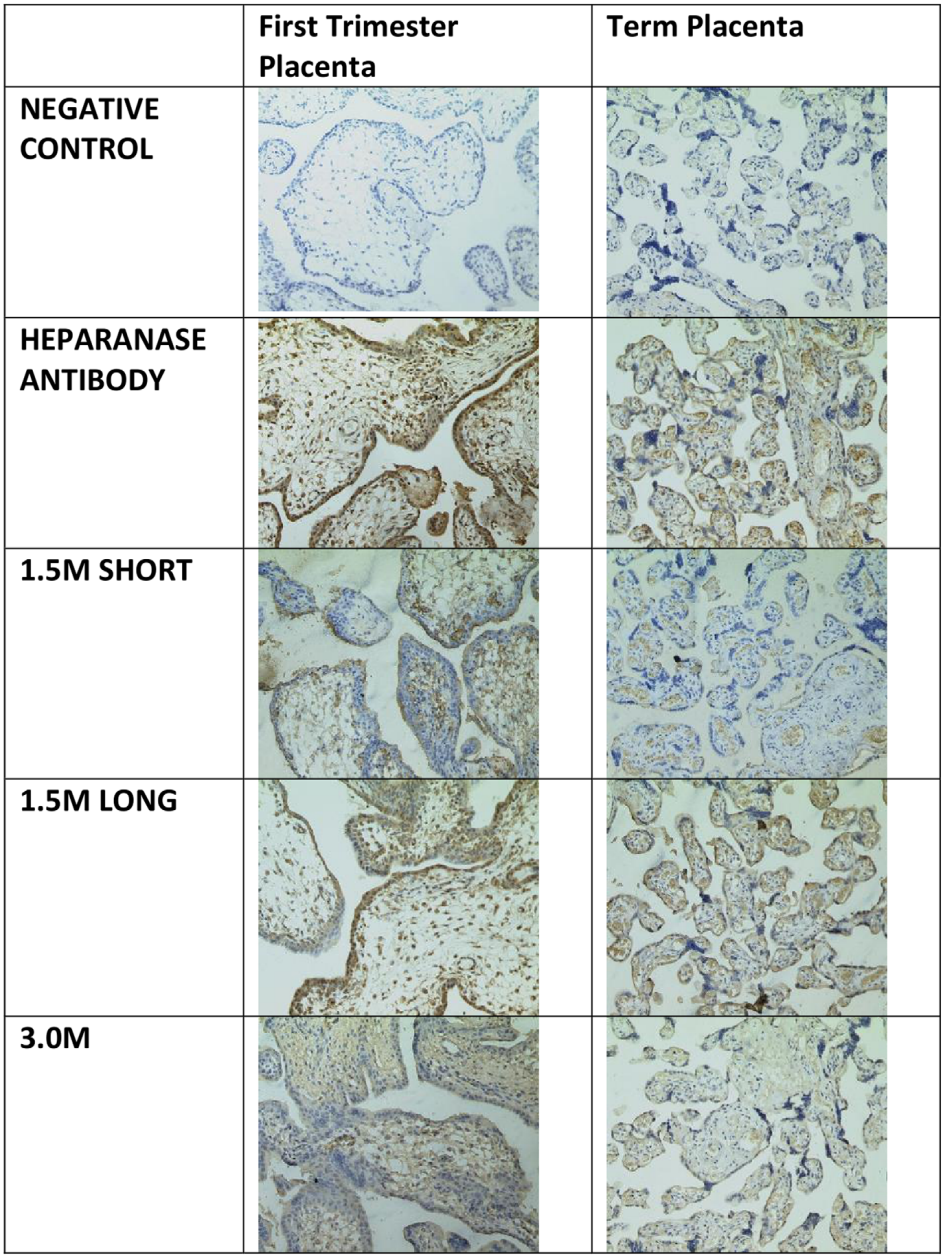
Immunohistochemistry (IHC) of paraffin embedded first trimester and term placenta tissue sections using heparanase polyclonal antibody, ‘1.5 M short’, ‘1.5 M long’ and ‘3.0 M’ aptamers, anti-cytokeratin-7 antibody plus a negative control mouse antibody.

**Figure 7:**
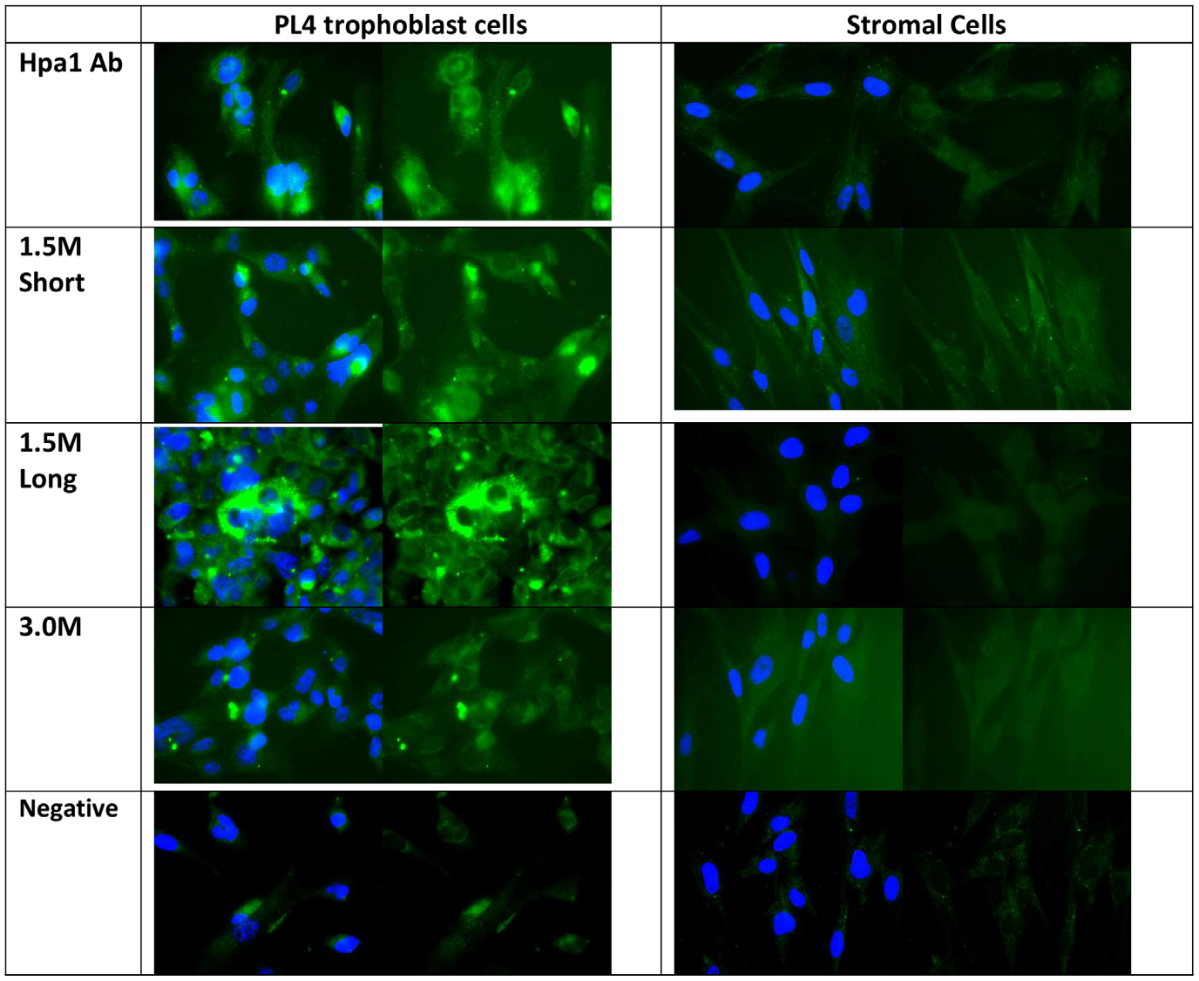
Fluorescence (green) of PL4 and stromal cells stained with heparanase polyclonal antibody, ‘1.5 M short’, ‘1.5 M long’ and ‘3.0 M’ aptamers plus a negative control. Nuclei were stained blue.

To test for recognition and specificity of the aptamers in comparison with the heparanase polyclonal antibody, paraffin-embedded placental tissue was sectioned and subjected to immunohistochemistry (figure 6), and immunofluorescent labelling was performed on different invasive cell lines (figure 7).

In first trimester placenta stained with polyclonal anti-heparanase antibody, staining was seen in the nuclei and cytoplasm of the cytotrophoblast-syncytiotrophoblast bilayer lining the intervillous space of the chorionic villi, and in endothelial cells of the developing blood vessels. No staining was observed when a mouse antibody was used as a negative control. Of the aptamers, 1.5 M short stained the cytoplasm of the outer syncytiotrophoblasts and 1.5 M long exhibited a staining pattern almost identical, although less intense than the antibody. 3.0 M did show staining, although more uniformly throughout the stroma and not in the same pattern as the antibody, which suggests non-specific staining.

In term placenta, heparanase antibody staining remains localised to syncytiotrophoblasts, and blood vessels. The negative control is unstained. 1.5 M long aptamer shows an identical staining pattern and intensity to the heparanase antibody. 1.5 M short stains fetal capillaries and 3.0 M shows a low level of stromal staining, which suggests once again non-specific binding.

PL4, a first trimester immortalised trophoblast cell line, when fluorescently labelled with the anti-heparanase antibody (figure 7), showed high levels of heparanase throughout the cytoplasm and in the nucleus of the cells, with some notable variation in intensity between adjacent cells. 1.5 M short and 1.5 M long aptamers showed similar or higher heparanase levels and staining patterns to that of the antibody. 3.0 M was also able to recognise heparanase in PL4 cells, but to a lesser extent than its counterparts. Stromal cells isolated from placental villous mesenchyme, contain a low level of heparanase both in tissue sections and in culture, as detected by specific antibody; aptamer binding patterns were entirely consistent with this level of expression. Interestingly, 1.5 M short aptamer has stained the cytoplasm of the cells to a higher degree than both the 1.5 M long and 3.0 M aptamers and the heparanase antibody.

### Aptamer Species Inhibit Heparanase in a Matrigel Cell Invation Assay

**Figure 8:**
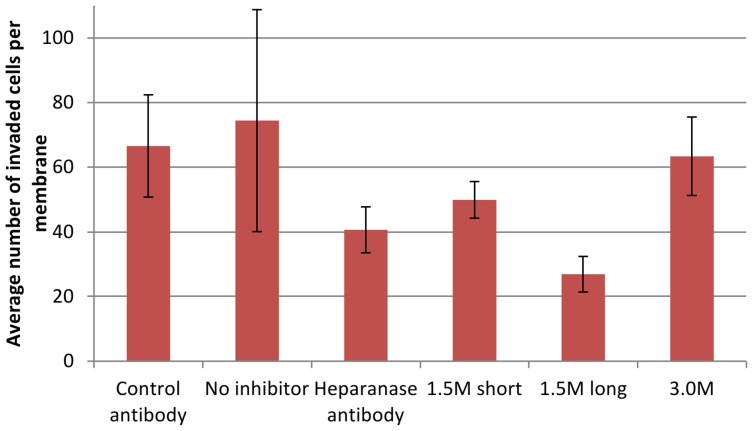
OC MZ-6 cells cultured in medium supplemented with anti-heparanase antibody, 1.5 M short, 1.5 M long and 3.0 M aptamers. The negative controls were represented as no inhibitor and a control mouse antibody. Figures are representative of the average of triplicate experiments and error bars show standard deviation of the mean.

The Matrigel invasion assay was performed to investigate whether binding of aptamers to heparanase inhibited the enzyme’s function of hydrolysing heparan sulphate proteoglycans (figure 8). This experiment was conducted using ovarian carcinoma cell line, OC MZ-6; a cell line previously shown to contain heparanase in immunofluorescence experiments [21]. A polyclonal anti-heparanase antibody that had also previously been used in this assay demonstrating inhibitory effects [21] was used as a positive control, and showed once more that significantly decreased numbers of cells had invaded the Matrigel, with 40.6±7.13 cells compared to no inhibitor at 74.4±34.28 (p = 0.0164) and compared with a negative control antibody at 66.6±15.77 (p = 0.0008). Anti-heparanase aptamers 1.5 M short, 1.5 M long and 3.0 M were all used as potential inhibitors (at a concentration of 1 µM) and of these, 1.5 M long significantly outperformed the heparanase antibody in its inhibitory function with 26.9±5.49 cells compared with no inhibitor (p = 0.0032) and with the negative control antibody (p<0.0001). 1.5 M short aptamer also demonstrated inhibition of invasion, though not at a significant level, and aptamer 3.0 M demonstrated little or no inhibiton of heparanase activity. These results suggest that the 1.5 M long aptamer is a significant inhibitor of cell invasion in this model.

## Discussion

Aptamers were successfully selected against the active heterodimer form of heparanase using an optimised, modified SELEX protocol based on interruption of the interactions formed between protein and aptamer by high salt content. Aptamers generated from 1.5 M NaCl and 3.0 M NaSCN elutions each suggested one prominent species based on sequence homology. Based on their predicted structures using Mfold structure predicting software, the aptamers were truncated to remove any nucleotides not implicated in structure formation for downstream considerations, such as cost and improved tissue penetration, before carrying out preliminary binding experiments. The binding data obtained from ELISA, QCM and fluorescence quenching titrations confirmed that the aptamers bind heparanase with high affinity, possibly close to two of its six tryptophan residues, but at a different site to that of the antibody, which is still able to bind heparanase simultaneously with the aptamers. Equilibrium dissociation constants for the aptamers generated to heparanase were calculated as 13.7 nM, 12 nM and 13 nM for 1.5 M short, long and 3.0 M respectively, which compares with high affinity aptamers generated by other groups for their targets [35], [45], [46], [47], [48]. Immunohistochemistry and immunofluorescence data suggest that aptamers 1.5 M short, long and 3.0 M are able to recognise and bind heparanase *in vitro* in the presence of other cell components and proteins. 1.5 M long and short aptamers showed the most consistent staining patterns respectively, compared with that of the antibody; in some cases exceeding the antibody in intensity and suggesting better recognition. As aptamers 1.5 M long and short originated from the same sequence and therefore share some structural features or binding nucleotides, these results were not totally unexpected. These results were consistent with those from the Matrigel invasion assay, where aptamer 1.5 M long outperformed the anti-heparanase antibody in inhibiting invasion of OC MZ-6 cells, suggesting a potential use as a heparanase inhibitor. Future experiments in other cell invasion or metastasis models will be necessary to determine the best inhibitor for *in vivo* studies and potentially the ideal aptamer length for inhibitory activity; a truncated aptamer may be favoured in cell and tissue-based studies as, due to its size, it may show increased tissue penetration. Although aptamer 3.0 M was eluted from the 3.0 M NaSCN wash and displayed high binding affinity in ELISA, QCM and fluorescence quenching, it did not perform as well in cell and tissue labelling studies or in the Matrigel invasion assay. Studies are now underway to further investigate the effect of the aptamer binding on both HS digestion and the non enzymatic roles of the protein. Other areas of future research are to map the specific binding regions of the aptamers on heparanase using truncated proteins, which may help to explain the lack of inhibitory action of the 3.0 M aptamer. Heparin-like molecules can exert anti-inflammatory effects and reduce leukocyte adhesion and trafficking, partly through their inhibition on heparanase activity [49], [50]. Future experiments will probe inflammatory model systems with specific heparanase DNA aptamers to address whether they can also influence the processes of immune cell diapedesis and extravasation. This paper demonstrates that aptamers can be selected to bind heparanase with high affinity and that these could be useful tools to examine the functional role of the protein and in diagnostic assay development or therapeutic application as heparanase inhibitors.

## Materials and Methods

### Selection Including Cloning and Sequencing

Aptamer library (5′–GGGAGACAAGAATAAACGCTCAA-25N-TTCGACAGGAGGCTCACAACAGGC-3′) (University of Nottingham, UK) was firstly amplified exponentially with 10 µM forward (5′–GGGAGACAAGAATAAACGCTCAA-3′) and reverse primer (5′-GCCTGTTGTGAGCCTCCTGTCGAA-3′ both from University of Nottingham, UK) to yield multiple species of double stranded DNA, which were then amplified using 350 µM forward primer only in a single stranded PCR, yielding multiple species of single stranded DNA aptamers. 150 nM human recombinant active heparanase protein (kindly supplied by Dr Edward McKenzie, University of Manchester, UK [51]) diluted in 100 mM salt solution, pH 6.5 (100 mM NaCl, 20 mM KCl, 5 mM MgCl_2_ all purchased from Sigma Aldrich Ltd, Gillingham, UK) was adsorbed to a Top Yield PCR tube (NUNC supplied by Fisher Scientific, Loughborough, UK) by overnight incubation at 4°C, after which any unbound heparanase was removed with a wash step using 100 mM salt solution. 100 µl amplified single-stranded library was incubated inside the heparanase-adsorbed tube, then the tube was sealed and gently agitated for one hour at room temperature. This step was repeated for a further 300 µl of amplified library so that the maximum aptamer species were in proximity to the heparanase molecules. Unbound and low-affinity bound species were removed by incubating 50 µl 0.2 M elution solution, pH 6.5 (0.2 M NaCl, 2.66 mM KCl, and 0.6 mM MgCl_2_) within the tube on a shaker for 5 minutes. The eluate was removed and retained. Elution of bound aptamers was achieved by using a salt gradient ranging from 300 mM to 1.5 M in 100 mM increments, with a final elution of 3.0 M NaSCN (Sigma Aldrich Ltd, Gillingham, UK), whilst retaining the eluates separately. The eluates were desalted using Microcons filters (Millipore, supplied by Fisher Scientific, Loughborough, UK) with a 3kDa molecular weight cut off, then amplified in a PCR to yield double-stranded DNA using 0.25 µM of each forward and reverse primer (a negative control, using water instead of DNA was also amplified at this stage). 10 µl from each eluate, plus the negative control and a 25bp DNA ladder were run on a 2% agarose gel to check for the presence of DNA aptamers. Fractions with the highest affinity species (from the highest concentrated salt elutions) were cloned into Top 10 chemically competent cells using pCR®2.1-TOPO® Vector from the TOPO® TA Cloning® Kit (Invitrogen, Paisley, UK). Aptamer-positive colonies were then sequenced by Macrogen Inc, Korea and the resulting sequences were analysed using Clustal [52] and Mfold structure prediction software [53], [54].

### ELISAs

187 nM 5′ biotinylated aptamers (Eurofins; Ebersberg, Germany) were immobilised to the wells of a Maxisorp ELISA plate (NUNC supplied by Fisher Scientific, Loughborough, UK) via 2.5 µg/ml NeutrAvidin® (Invitrogen, Paisley, UK) in PBS (10 mM phosphate buffer, 2.7 mM potassium chloride and 137 mM sodium chloride, pH 7.4, Sigma Aldrich Ltd, Gillingham, UK) overnight at 4°C. After the plate was washed three times with wash buffer (5% Tween 20 in PBS), sample wells were blocked with 10 mg/ml BSA (Sigma Aldrich Ltd, Gillingham, UK) in PBS for 20 minutes at room temperature. After three washes in wash buffer, heparanase was added to sample wells in series between 0 and 200 nM in assay diluent (0.1% Tween 20, 72 mM BSA in PBS), and incubated for one hour at room temperature. After a further three washes in wash buffer, polyclonal rabbit anti-heparanase IgG (kindly supplied by Professor Paul Brenchley, University of Manchester, UK) [21] was added at a concentration of 200 nM to all sample wells and incubated for one hour at room temperature. After three washes in wash buffer, 1/700 dilution of peroxidase-conjugated goat anti-rabbit secondary antibody (Sigma Aldrich Ltd, Gillingham, UK) was incubated in all sample wells for one hour at room temperature, after which, the wells were washed in wash buffer once again. Chromogen containing tetramethylbenzidine (TMB, Sigma Aldrich Ltd, Gillingham, UK) as substrate (100 mM Sodium acetate, 3 mM citric acid, 0.1% TMB in DMSO, 0.04% H_2_O_2_) was incubated in each well for 30 minutes, followed by addition of 10% H_2_SO_4_ to halt the reaction. The plate was read at 450 nm using a Bio-Tek EL808 Ultra Microplate Reader with Datamax software.

### Quartz Crystal Microbalance

Experiments were carried out according to Liss et al [55], using a research quartz crystal microbalance (QCM) from Maxtek Inc. Gold surfaces of each 5MHz crystal (Testbourne Ltd, Basingstoke, UK) were cleaned with ice-cold piranha solution (1 volume 30% H_2_O_2_ added to 4 volumes concentrated H_2_SO_4_). Crystals were immersed in ice-cold H_2_O, and then transferred to an oven to dry at 100°C for at least an hour. Activation of gold surfaces was achieved by adding 4 mg/ml 3,3′ dithiodipropionic acid (DSP) in water-free N,N-dimethylacetamide (DMA) (both from Sigma Aldrich Ltd, Gillingham, UK), sealing the crystal within a chamber and incubating for 15 minutes at room temperature. The crystals were washed three times with sterile PBS. 1 mg/ml streptavidin diluted in sterile PBS was deposited onto the crystal surface and crystals were sealed in a chamber and incubated overnight at 4°C. The crystals were washed five times with PBS, and then blocked by adding 0.025% BSA in PBS and incubating for one hour at room temperature. 5′ biotinylated aptamers, diluted to 2 µM in PBS were added after being heated to 95°C and cooled on ice, and incubated for one hour at room temperature. The crystals were washed well in PBS and used immediately.

To measure the binding of heparanase to the aptamers immobilised on the gold surface, the crystal was placed in the flow cell which was connected to a pump. Samples were drawn through into the flow cell in a stop-flow manner. PBS was run through the system for one minute on fast flow, then the pump was halted, the program and monitoring switched on, the capacitance adjusted, and the crystal left to equilibrate in the PBS contained within the flow cell for one hour or until the reading was stabilised. After pausing the program and taking care not to introduce any air into the system, human recombinant polymorphic heparanase diluted in PBS was added to the flow cell, using the pump to pull the liquid through very slowly, then the pump was halted and the program was resumed. When the readings had stabilised, the program was paused and the crystal was washed using the pump to pull 10 ml PBS through the system, the pump was ceased and the readings resumed. After the readings had settled, the program was paused and the crystal was regenerated with 10 ml 100 mM EDTA, then 3.0 M NaSCN (both from Sigma Aldrich Ltd, Gillingham, UK) before the experiment ended. The crystal was kept at 4°C in a chamber of PBS containing 0.02% sodium azide until the next experiment. The results, displayed as Δf_r_ (change in resonance frequency), are inversely related to the mass at the surface of the crystal (Δm) as explained in the Sauerbrey equation [55]. Hence, when mass is deposited at the crystal’s surface, the resonance frequency decreases.

### Fluorescence Quenching Experiments

Experiments were performed using a Horiba Jobin Yvon Fluoromax-P fluorimeter, with the sample in a 100 µl fluorescence cuvette. Emission spectra were acquired between 300 and 420 nm with an excitation wavelength of 280 nm and integration time of 1s, at 5 nm increments and at room temperature. After performing the acquisition on 250 nM heparanase in salt solution, aptamer was titrated to the following final concentrations in the cuvette, mixed and an acquisition taken immediately: 25 nM, 50 nM, 100 nM, 200 nM, 400 nM. Results were analysed using Microcal Origin 6 and the equilibrium association constant (K_A_) determined using the equation described by Missailidis and Brady [56].

### Cell Culture and Tissue Collection

Immunohistochemistry, immunofluorescence and Matrigel invasion studies were carried out within the Maternal and Fetal Health Research Centre at The University of Manchester. All media and supplements were purchased from Invitrogen Ltd (Paisley, UK).

First trimester placental samples from women undergoing elective terminations of pregnancy between 8–12 weeks gestation and term placental samples were obtained from St. Mary’s Hospital, Manchester. Written informed consent was obtained from all patients and ethical approval was obtained from Central Manchester Local Research Ethics Committee (03/CM/031).

Cell lines were cultured to 80% confluency for the following experiments and media were supplemented with 10% FBS, L-glutamine (2 mM), penicillin (100 IU/ml) and streptomycin (100 µg/ml). Placental stromal cells (first trimester) were cultured in 50% DMEM 50% Ham’s F12 supplemented with 1% non-essential amino acids and first trimester transformed PL4 trophoblasts were cultured in Ham’s F10 medium. Ovarian carcinoma cell line OC MZ-6 was cultured in DMEM containing 10 mM HEPES, 116 mg/L arginine and 36 mg/L asparagine.

### Immunoperoxidase Staining of Tissue

Wax embedded samples of first trimester and term placenta were sectioned at 5 µm. Slides were warmed for 10 min at 60°C to soften the wax, then deparaffinised in xylene and alcohol, and rinsed in tap water. Slides were immersed in citrate buffer (2.94g sodium citrate in 1l of dH_2_O, pH 6.0, with 500 µl Tween 20) and microwaved at high temperature for a total of 10 min. After cooling, endogenous peroxide activity was blocked by placing slides in methanol containing 0.4% (v/v) HCl and 0.5% (v/v) H_2_O_2_ for 30 min. After rinsing in running water, the slides were washed for 5 min in TBS. Sections were blocked for 30 min with 5% BSA in TBS, and then primary antibody/5′ biotinylated aptamers were added in TBS (1∶100 dilutions for anti-heparanase and anti-cytokeratin 7, neat for control mouse antibody and 500 nM for all aptamers) and left overnight at 4°C in humidity chamber. After washing slides for 3×5 min in TBS, secondary antibodies were incubated at 1∶200 dilutions for 1h at room temperature to relevant sections and aptamer-labelled sections were incubated with TBS only. Slides were washed three times for 5 min each in TBS, and then avidin peroxidase was added to all sections at 5 µg/ml in TBS and incubated at room temperature for 1h, then washed off with TBS. 3,3′-Diaminobenzidine tetrahydrochloride (DAB) was used to develop the avidin peroxidase (280 ml TBS +20 ml DAB. 45 µl H_2_O_2_ was added before use). Slides were washed in tap water and counterstained with Meyers’ haematoxylin for 5 min, then once more washed with tap water. Slides were dipped in acid alcohol solution to remove excess stain for five seconds and then immersed in tap water for 5 min. Slides were dehydrated and mounted with large coverslips using xylene alternative mountant.

### Immunofluorescence

PL4 and placental stromal cells were fixed in 4% paraformaldehyde for 15 min, permeabilised by incubating them in 0.1% Triton X100 in PBS for 10 min, then washed in PBS. Primary antibody/biotinylated aptamers in PBS were added and incubated for 1h at room temperature (1∶100 dilutions for anti-heparanase and anti-cytokeratin 7, neat for control mouse antibody and 1∶200 dilutions for all aptamers). After 3×5 min washes in PBS, secondary antibodies were added at 1∶200 to Hpa1 and control mouse labelled coverslips only and incubated for 1h at room temperature (aptamer-labelled coverslips were left in PBS). After washing in PBS (3×5 min), streptavidin-FITC conjugate was added to all coverslips at a 1∶50 dilution of stock and incubated for 1h at room temperature underneath a cover to preserve fluorescence. After a further 3 PBS washes, coverslips were blotted on a soft tissue and mounted using Vectashield mounting medium (Vector), containing propidium iodide.

### Matrigel Invasion Assay Method

10 mm tissue culture inserts with an 8 µm pore size (Nunc, Fisher Scientific, UK) were placed into each experimental well of a sterile 24-well plate (Nunc, Fisher Scientific, UK). One coverslip per experiment was sterilised in IMS to be used as an indicator of cell confluency throughout the experiment. 20 µl Matrigel (BD Discovery Labware, Bedford, MA, USA) previously diluted to working concentration; (1∶8 in serum-free medium) was spread over each insert then left to dry in the incubator for 30 min.

500 µl medium was added around the edge of each insert and then 400 µl medium plus 6×10^4^ cells in 100 µl medium was placed on the top of the Matrigel and left to adhere for 1h in the incubator. The medium from both the insert and the well was removed then replaced with aptamer/antibody/control solution (all at 1∶100 dilutions in fresh medium); 500 µl each into the insert and the well, and the plate was incubated for 24h. The cells were fixed in 4% paraformaldehyde for 15 min and permeabilised in 0.1% Triton X100 for 10 min, then stored in PBS @4°C until they were ready to be stained. The insides of each membrane were wiped with a cotton bud to remove all cells, leaving only the cells on the underside, which were then stained with freshly filtered haematoxylin. After a PBS wash, the inserts were plunged into a beaker of hot water to activate the stain. The insides of each membrane were once again wiped with a cotton bud and removed from the transwell, then placed on slides and mounted. The slides were viewed under light microscope at ×10 magnification, eight to ten representative areas were selected and the cells counted. Data were represented as the mean ± standard deviation and an unpaired two-tailed t-test was conducted to assess the significance of the results.
