# Correction: Development of Novel Single-Stranded Nucleic Acid Aptamers against the Pro-Angiogenic and Metastatic Enzyme Heparanase (HPSE1)

**DOI:** 10.1371/annotation/48b14416-bb2e-422f-89db-cf7f81df61aa

**Published:** 2012-09-28

**Authors:** Suzanne C. Simmons, Edward A. McKenzie, Lynda K. Harris, John D. Aplin, Paul E. Brenchley, Maria N. Velasco-Garcia, Sotiris Missailidis

There was an error in the second affiliation. The correct affiliation is:

2 Manchester Institute of Biotechnology, University of Manchester, Manchester, Metropolitan County of Greater Manchester, United Kingdom. 

